# Skin secretions of Leptodactylidae (Anura) and their potential applications

**DOI:** 10.1590/1678-9199-JVATITD-2023-0042

**Published:** 2024-02-19

**Authors:** Juan F. C. Carrillo, Amanda Galdi Boaretto, Diego J. Santana, Denise Brentan Silva

**Affiliations:** 1 Program in Ecology and Conservation, Institute of Biosciences, Federal University of Mato Grosso do Sul, Campo Grande, MS, Brazil.; 2 Laboratory of Systematics and Biogeography of Amphibians and Reptiles (Mapinguari), Institute of Biosciences, Federal University of Mato Grosso do Sul, Campo Grande, MS, Brazil.; 3 Laboratory of Natural Products and Mass Spectrometry (LaPNEM), Faculty of Pharmaceutical Sciences, Food and Nutrition (FACFAN), Federal University of Mato Grosso do Sul, Campo Grande, MS, Brazil.

**Keywords:** Antimicrobial peptides, Peptides, Amines, Antibiotic resistance

## Abstract

The skin of anuran species is a protective barrier against predators and pathogens, showing also chemical defense by substances that represent a potential source for bioactive substances. This review describes the current chemical and biological knowledge from the skin secretions of Leptodactylidae species, one of the most diverse neotropical frog families. These skin secretions reveal a variety of substances such as amines (12), neuropeptides (16), and antimicrobial peptides (72). The amines include histamine and its methylated derivatives, tryptamine derivatives and quaternary amines. The peptides of Leptodactylidae species show molecular weight up to 3364 Da and ocellatins are the most reported. The peptides exhibit commonly glycine (G) or glycine-valine (GV) as C-terminal amino acids, and the most common N-terminal amino acids are glutamic acid (E), lysine (K), and valine (V). The substances from Leptodactylidae species have been evaluated against pathogenic microorganisms, particularly *Escherichia coli* and *Staphylococcus aureus*, and the most active peptides showed MIC of 1-15 µM. Furthermore, some compounds showed also pharmacological properties such as immunomodulation, treatment of degenerative diseases, anticancer, and antioxidant. Currently, only 9% of the species in this family have been properly studied, highlighting a large number of unstudied species such as an entire subfamily (Paratelmatobiinae). The ecological context, functions, and evolution of peptides and amines in this family are poorly understood and represent a large field for further exploration.

## Background

Amphibian skin has a wide range of physiological functions, including defense against predators and microorganisms through the secretion of chemical substances, gas exchange, and water balance [1, 2]. These animals have a great variety of predators, such as mammals, birds, snakes, and spiders, resulting in a diverse array of defensive substances [1]. Alkaloids from poison frogs and toads (e.g. Dendrobatidae and Bufonidae), for example, can be noxious to predators, while proteins from Bufonidae, Hylidae, Leptodactylidae, and Odontophynidae can reduce palatability [3-6]. Amphibians are exposed to diverse environmental conditions, and their skin must protect them from microorganisms found in water, soil, and air [7-9]. As a result, they rely on chemical defenses, which can be peptide-based and supplemented by other substances, such as alkaloids. [10-12].

The metabolites associated with chemical defense are generally stored in the epithelial glands [9]. The most common glands present in amphibian skin are mucus and granular glands, although some species carry specialized glands with particular functions [13, 14]. Mucus glands are specific for mechanical functions, such as lubrication in aquatic environments and humidification in terrestrial environments [15]. Mucus is primarily commonly related to mechanical functions, lubrication, and humidification, but it also plays a role in water balance and gas exchange, exhibiting antimicrobial properties occasionally [1, 15]. Granular glands, on the other hand, are more specialized in defense against predators and microbial infections, which accumulate peptides, alkaloids, and amines, exhibiting various biological properties, such as prevention of microbial infections [16-18].

Due to the natural exposure to pathogens and the species diversity of amphibians, the study of skin secretions represents a great potential to discover new bioactive molecules [1, 16, 19, 20]. This represents a great opportunity to counter public health issues, such as bacterial infections exacerbated by resistant strains and the ability of bacteria to evade therapeutic antibiotics through biofilm formation. Bacterial infections also carry high morbidity and mortality rates, estimating an increase in deaths that may surpass cancer deaths in 2050 [21]. This estimation has been exacerbated by the drug-resistant bacteria, in particular *Staphylococcus aureus* and their resistant strains to methicillin (MRSA), beta-lactams, and carbapenems [22-24]. This health problem was intensified by the COVID-19 pandemic due to the irrational use of antibiotics [25, 26], as well as bacterial biofilms with recurrent infections [27]. Although previous reviews have presented the chemical composition of the skin secretion of anurans [11, 12], topics related to antimicrobial activities and ecological functions have been overlooked. Besides, other anuran families, such as Bufonidae and Dendrobatidae, overshadow leptodactylids species. Here, we review the current knowledge about the skin secretion of Leptodactylidae species and their potential applications. We restricted our research to the current species of the Leptodactylidae following Frost [20]. As the family systematics and taxonomy have been continuously modified [28-31], we update data of the species name to avoid confusion about chemistry, systematics, and chemotaxonomy (Additional file 1). Species without information about collection locality or with uncertainty about species determination were updated using synonymy by Frost [20].

Therefore, this review was based on previous chemical and biological studies from Leptodactylidae (Anura) focused on skin peptides and other substances, especially against pathogenic microorganisms, such as the antimicrobial peptides (AMPs), in addition to the ecology and evolution of the explored substances. The antimicrobial peptides (AMPs) of anurans from skin secretions have been targeted in several studies. They have also shown antiviral properties against several types of viruses, such as dengue, influenza A (H1N1 and H5N1), human immunodeficiency virus (HIV), human papillomavirus (HPV), herpes simplex, Zika virus, and SARS-CoV-2. Their antiviral mechanism actions have been described by interaction or disruption of capsid virus, suppression of gene expression, modulation of the immune system, blocking of the virus entry into cells, and inhibition of viral replication or synthesis of proteins [32].

### Leptodactylid frogs

**Figure 1.**
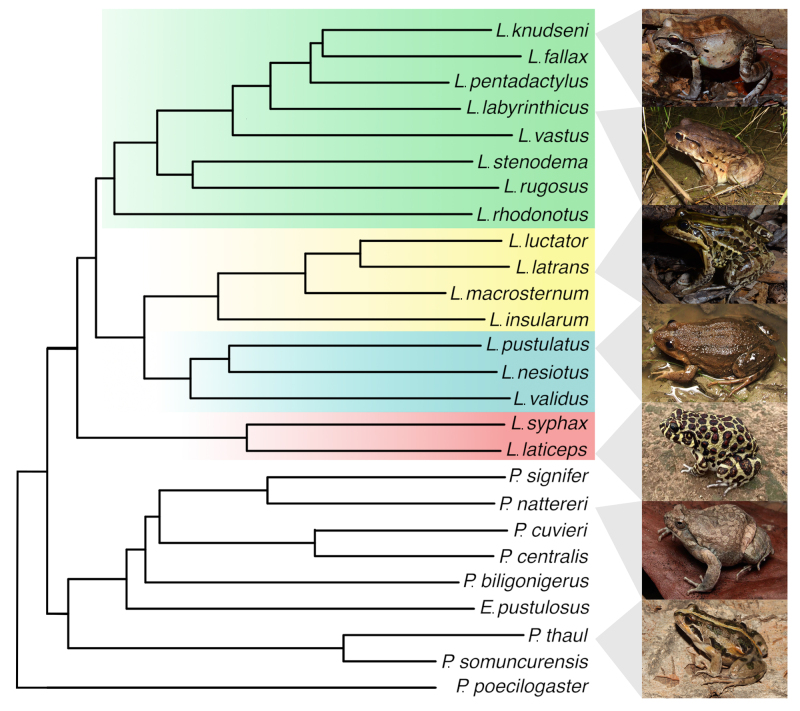
Phylogenetic tree of Leptodactylidae species with studies of skin secretion. *Leptodactylus knudseni* (Photo by Diego Santana), *L. fallax*, *L. pentadactylus*, *L. labyrinthicus* (Photo by Diego Santana), *L. vastus*, *L. stenodema*, *L. rugosus*, *L. rhodonotus*, *L. luctator*, *L. latrans* (Photo by Diego Santana), *L. macrosternum*, *L. insularum, L. pustulatus* (Photo by Diego Santana), *L. nesiotus*, *L. validus*, *L. syphax*, *L. laticeps* (Photo by Hugo Cabral), *Physalaemus signifier*, *P. nattereri* (Photo by Diego Santana), *P. cuvieri*, *P. centralis*, *P. biligonigerus*, *Engystomops pustulosus*, *Pleurodema thaul* (Photo by Diego Baldo), *P. somuncurensis*, *Paratelmatobius poecilogaster* (outgroup). Colours represent species groups of *Leptodactylus*: *L. pentadactylus* group (green), *L. latrans* group (yellow), *L. melanonotus* (blue), and *L. fuscus* group (red).

Leptodactylidae Werner, 1896 is one of the most diverse and widely distributed frog families in the neotropical region [20], and it presents large potential to research new bioactive compounds. Frogs in this family can be found from Mexico (Sonora) throughout Central and South America to Argentina and Brazil, including northern Antilles [20]. Leptodactylidae comprises more than 230 species (Figure 1), distributed in three monophyletic subfamilies: Leiuperinae, Leptodactylinae, and Paratelmatobiinae [33]. Leiuperine has 101 species distributed in five genera (*Edalorhina*, *Engystomops*, *Physalaemus*, *Pleurodema,* and *Pseudopaludicola*). Leptodactylinae shows 118 species distributed in four genera (*Adenomera*, *Hydrolaetare*, *Leptodactylus,* and *Lithodytes*), while Paratelmatobiinae represents 15 species in four genera (*Crossodactylodes*, *Cochran*, *Paratelmatobius*, *Rupirana* and *Scythrophrys*) [20, 33]. *Leptodactylus,* the most diverse genus in the family, includes 84 species arranged in four species groups (*L. fuscus*, *L. latrans, L. melanonotus,* and *L. pentadactylus*)*,* according to molecular phylogeny, reproductive modes, anatomy, and additional behavioral characteristics [28, 34].

Most species of Leptodactylidae are terrestrial, can be found in open formations in forested areas, and feed in leaf litter or close to temporary ponds [28]. Although these species can commonly habit lowland ecosystems, several of them can reach high mountainous areas over 1200 meters above sea levels (m.a.s.l.), such as *Leptodactylus fragilis*, *L. fuscus*, *L. savagei*, and *L. ventrimaculatus* [35]. Further, *L. colombiensis* can reach 2800 m.a.s.l. in the Colombian Cordillera Oriental [35]. Additionally, several endemic species are from high-altitude ecosystems (e.g. *Leptodactylus oreomantis* and *Physalaemus rupestris*) [36, 37].

Representative species (Figure 1) for the study of skin metabolites from Leptodactylidae showed extensive distributions such as *Leptodactylus knudseni* (Bolivia, Brazil, Colombia, Ecuador, French Guiana, Guyana, Peru, Suriname, and Venezuela), *L. fallax* (Jamaica and Puerto Rico), *L. pentadactylus* (Brazil, Colombia, Ecuador, French Guiana, Guyana, Peru, Suriname, and Venezuela), *L. labyrinthicus* (Argentina, Brazil and Paraguay), *L. vastus* (Bolivia and Brazil), *L. stenodema* (Brazil, Colombia, Ecuador, French Guiana, Guyana, Peru, and Suriname), *L. rugosus* (Brazil, Guyana, and Venezuela), *L. rhodonotus* (Bolivia, Brazil, Colombia, and Peru), *L. fallax* (Jamaica and Puerto Rico), *L. luctator* (Argentina, Bolivia, Brazil, and Uruguay), *L. latrans* (Brazil), *L. macrosternum* (Argentina, Bolivia, Brazil, Colombia, French Guiana, Guyana, Paraguay, Peru, Suriname, Trinidad and Tobago, Uruguay, and Venezuela), *L. insularum* (Colombia, Costa Rica, Panama, Trinidad and Tobago and Venezuela), *L. pustulatus* (Brazil), *L. nesiotus* (French Guiana, Guyana, Suriname, Trinidad and Tobago), *L. validus* (Brazil, Colombia, Guyana, Suriname, Trinidad and Tobago, and Venezuela), *L. syphax* (Bolivia, Brazil, and Paraguay), *L. laticeps* (Argentina, Bolivia, and Paraguay), *Physalaemus nattereri* (Bolivia, Brazil, and Paraguay), *P. cuvieri* (Argentina, Bolivia, Brazil, Guyana, Paraguay, Uruguay, and Venezuela), *P. centralis* (Bolivia, Brazil, and Paraguay), *P. bibigonigerus* (Argentina, Bolivia, Brazil, Paraguay, and Uruguay), and *Engystomops pustulatus* (Ecuador and Peru) [20]. However, there are species with restricted distributions, such as *Physalaemus. signifier* (Brazil), *Pleuroderma thaul* (Argentina), and *P. sumoncurensis* (Argentina) [20].

## Skin metabolites of Leptodactylidae

The main substances described in the skin secretion of Leptodactylidae species are amines and peptides (Tables 1 and 2). These compounds were 12 amines from 15 species of one genus and 88 peptides classified as neuroactive peptides (16) and antimicrobial peptides (72) from 25 species of four genera. *Leptodactylus* is the genus with a higher number of peptides described.

### Amines

Amines have been described from Leptodactylidae species as summarized in Table 1. The first isolated substance from the skin of a Leptodactylidae species was the biogenic amine leptodactyline, which was isolated in 1959 from *Leptodactylus luctator* under the name of *Leptodactylus ocellatus* [38]. Biogenic amines are nitrogenous organic molecules with low molecular weight yielded from the decarboxylation of amino acids or amination and transamination of aldehydes or ketones. These biogenic amines, which are associated with several biological activities, have also been identified in plants, animals, and microorganisms [39]. 

**Table 1.** Amines from the skin secretion of the Leptodactylidae species.

Amine	Species	Chromatographic analysis	Reference
5-hydroxytryptamine (5-HT) (C_10_H_12_N_2_O, MW 176.2)	*Leptodactylus labrosus*	ACC	[40]
	*Leptodactylus labyrinthicus*	PC	[41,42]
	*Leptodactylus labyrinthicus*	ACC	[40]
	*Leptodactylus laticeps*	PC	[40-42]
	*Leptodactylus melanonotus*	PC	[41,42]
	*Leptodactylus pentadactylus*	PC	[40-42]
	*Leptodactylus petersii*	PC	[41,42]
	*Leptodactylus podicipinus*	PC	[41,42]
	*Leptodactylus rhodonotus*	PC	[40,41]
	*Leptodactylus vilarsi*	ACC	[40]
	*Leptodactylus stenodema*	ACC	[40]
6-Methylspinaceamine (C_7_H_11_N_3_, MW 137.2)	*Leptodactylus labyrinthicus*	PC	[41,42]
Bufotenidine (C_13_H_18_N_2_O, MW 218.3)	*Leptodactylus labrosus*	ACC	[40]
	*Leptodactylus melanonotus*	PC	[41,42]
	*Leptodactylus pentadactylus*	PC	[41,42]
		ACC	[40]
	*Leptodactylus petersii*	PC	[41,42]
	*Leptodactylus podicipinus*	PC	[41,42]
	*Leptodactylus rhodonotus*	PC	[41]
		ACC	[40]
	*Leptodactylus stenodema*	ACC	[43]
	*Leptodactylus vilarsi*	ACC	[40]
Candicine (C_11_H_18_NO^+^, MW180.3)	*Leptodactylus pentadactylus*	PC	[41,42]
Dehydrobufotenine (C_12_H_15_N_2_O^+,^ MW 203.3)	*Leptodactylus stenodema*	ACC	[43]
Histamine (C_5_H_9_N_3_, MW 111.1)	*Leptodactylus labyrinthicus*	PC	[41,42]
		ACC	[40]
	*Leptodactylus laticeps*	PC	[41,42]
		ACC	[40]
	*Leptodactylus pentadactylus*	PC	[41,42]
		ACC	[40]
	*Leptodactylus stenodema*	ACC	[43]
	*Leptodactylus vilarsi*	ACC	[40]
Leptodactyline (C_11_H_18_NO^+^, MW 180.3)	*Leptodactylus bolivianus*	PC	[41,42]
	*Leptodactylus bufonius*	PC	[41]
	*Leptodactylus labrosus*	ACC	[40]
	*Leptodactylus labyrinthicus*	PC	[41,42]
		ACC	[40]
	*Leptodactylus laticeps*	PC	[41,42]
		ACC	[40]
	*Leptodactylus latinasus*	PC	[38]
			[41,42]
	*Leptodactylus macrosternum*	PC	[41,42]
	*Leptodactylus melanonotus*	PC	[41,42]
	*Leptodactylus pentadactylus*	PC	[41,42]
		ACC	[40]
	*Leptodactylus petersii*	PC	[41,42]
	*Leptodactylus podicipinus*	PC	[41,42]
	*Leptodactylus rhodonotus*	PC	[41,42]
		ACC	[40]
	*Leptodactylus stenodema*	ACC	[38]
	*Leptodactylus vilarsi*	ACC	[40]
*N,N*-Dimethylhistamine (C_7_H_13_N_3_, MW 139.20)	*Leptodactylus labyrinthicus*	PC	[41,42]
*N*-Methyl-5-hydroxytryptamine (C_11_H_14_N_2_O, MW 190.2)	*Leptodactylus pentadactylus*	ACC	[40]
	*Leptodactylus stenodema*	ACC	[38]
	*Leptodactylus vilarsi*	ACC	[40]
	*Leptodactylus melanonotus*	PC	[41]
	*Leptodactylus petersii*	PC	[42]
*N*-Methylhistamine (C_6_H_11_N_3_, MW 125.2)	*Leptodactylus labyrinthicus*	PC	[41,42]
Spinaceamine (C_6_H_9_N_3_, MW 123.2)	*Leptodactylus labyrinthicus*	PC	[41,42]
	*Leptodactylus laticeps*	PC	[41,42]
Tyramine (C_8_H_11_NO, MW 137.2)	*Leptodactylus pentadactylus*	PC	[41,42]

MW: Molecular weight. PC: Paper Chromatography. ACC: Alumina Chromatography column.

**Figure 2.**
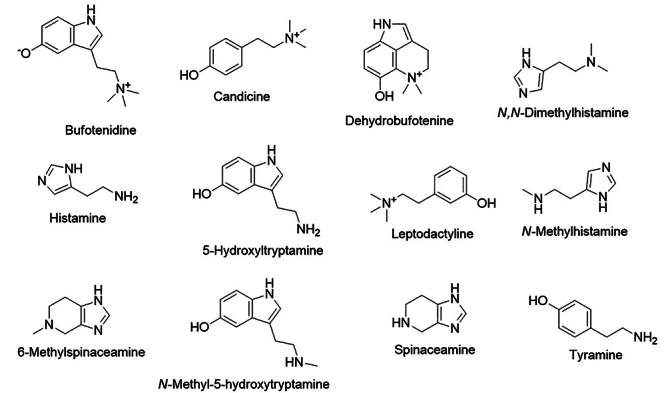
Chemical structures of the amines of Leptodactylidae species.

Erspamer (1971) classified the amines of amphibians into three groups: indole alkylamines, imidazole alkylamines, and hydroxyphenyl alkylamines, and all of them were registered from *Leptodactylus* spp. [1, 41, 44]. Leptodactyline (Figure 2) was the first *m*-hydroxyphenyl alkylamine described in animals, and among its functions are: the paralyzation of skeletal muscle, the induction of ganglion stimulation, and the nicotinic actions [45]. This amine has been registered in several other species of *Leptodactylus* (Table 1, Figure 2). 

Candicine (Figure 2) is another hydroxyphenyl alkylamine that was first isolated from plants of the family Cactaceae, and it is also found in anuran *Leptodactylus pentadactylus.* This amine has similar effects observed for leptodactyline in mammals but with lower activity [44]. 

Indole alkylamines found in *Leptodactylus* are 5-hydroxytryptamine (5-HT) and its *N*-methylated derivatives (Table 1). These compounds are also reported from other species of families Ascaphidae, Ceratophryidae, Hylidae, Pelobatidae, Phyllomedusidae, Ranidae, and Rhinodermatidae [46]. Bufotenidine, an indole alkylamine, was initially isolated from European *Bufo vulgaris*, but it was also found in several species of *Bufo* and other families and genera [47], including species from Leptodactylidae (Table 1). 

The amines belonging to the imidazole alkylamines class described in Leptodactylidae include histamine and spinaceamine and their derivatives. Histamine and its derivates have been reported in *Leptodactylus* (Table 1), and induce cardiac stimulation and vasoconstriction, comparable to the stimulation effects of adrenaline in mammals [47,48]. Besides, some of these amines are also present in other anurans from Bufonidae, Hylidae, Telmatobiidade, Alsodidae, Odontophrynidae, Myobatrachidae, Microhylidae, Ranidae, Pipidae, Heleophrynidae, and Hyperoliidae [46, 49]. They are also reported only in the genus *Leptodactylus* of Leptodactylidae (Table 1). Spinaceamine is reported only from *L. laticeps* and *L. labyrinthicus* (Figure 1) [50]. Tyramine is a common amine described in both animals and plants [51], but it has been reported only for the Anura *L. pentadactylus* (Table 1).

### Peptides

The peptides are also a large group of substances described in the skin secretions of Leptodactylidae, mainly antimicrobial peptides (AMPs). Peptides are long chains of amino acids linked by a peptide bold [52]. Generally, frog peptides are cationic, varying 8 to 48 amino acid residues with several hydrophobic amino acids and predominant conformation of amphipathic α-helix [53]. Currently, over 80 peptides have been described from Leptodactylidae species (Table 2). For example, somuncurin-3 (DDGEEEAESEEANPEENTEGEKKKKCRRRKGSKLLRRCRGVKI-NH₂) is the greater and (Val1, Thr6, des-Arg9)-Bradykinin (VPPGFTPF) is the smallest peptide, which was described from *Pleurodema somuncurense* and *Physalaemus nattereri,* respectively (Table 2). 

**Table 2.** Peptides from the skin secretion of the Leptodactylidae family.

Species	Type	Peptide	Extraction	Technique*	Sequence	MW	Tmass (Esmass)	Reference
*Engystomops pustulosus*	NP	Physalaemin	SOE	ACC	EADPDKFYGLM-NH₂	1284.6	-	[54]
	AMP	Tigerinin-1EP	NI	HPLC	GCKTYLIEPPVCT	1424.7	1421.7	[55]
	AMP	pustulosin-1	NI	HPLC	FWKADVKEIGKKLAAKLAEELAKKLGEQ	3141.6	3141.8	[55]
	AMP	pustulosin-2	NI	HPLC	FWKADVKEIGKKLAAKLAEELAKKLGEE	3142.6	3142.8	[55]
	AMP	pustulosin-3	NI	HPLC	DWKETAKELLKKIGAKVAQVISDKLNPAPQ	3318.7	3318.9	[55]
	AMP	pustulosin-4	NI	HPLC	DWKADAKDILKKIGAKIAQVISDKLNPAPQ	3274.6	3274.8	[55]
*Leptodactylus fallax*	-	LASP	NI	HPLC	GLWDDLKAAAKKVVSSLASAAIEKL-NH	2583.5	2513.9	[56]
	AMP	Ocellatin-F1/Fallaxin	NI	HPLC	GVVDILKGAAKDIAGHLASKVMNKL-NH₂	2547.5	2549	[57]
*Leptodactylus insularum*	AMP	Ocellatin-1I	NI	HPLC	GLLDLLKGAGKGLLTHLASQIa	2117.3	2117.3 (2117.3)	[58]
	AMP	Ocellatin-1I (1-16)	NI	HPLC	GLLDLLKGAGKGLLTH	1605.0	1606.0 (1606.0)	[58]
	AMP	Ocellatin-2I	NI	HPLC	GLLDFFKGAGKELLTHLASQIa	2257.2	2257.2 (2257.3)	[58]
	AMP	Ocellatin-2I (1-16)	NI	HPLC	GLLDFFKGAGKELLTH	1745.0	1746.0 (1746.0)	[58]
	AMP	Ocellatin-3I	NI	HPLC	GVIDILKSLGKNILTNLASKLSDNTA	2697.5	2698.5 (2698.6)	[58]
*Leptodactylus knudseni*	AMP	Ocellatin-K1	MS	HPLC	GVVDILKGAAKDLAGHLASKVMNKL	2547.5	2547.65	[59]
*Leptodactylus labrosus*	NP	Caerulein-like peptide	SOE	ACC	-	-	-	[40]
*Leptodactylus labyrinthicus*	AMP	Ocellatin-F1/Fallaxin	SS	HPLC	GVVDILKGAAKDIAGHLASKVMNKL-NH₂	2547.5	2545.4 (2546.5)	[60]
	AMP	Ocellatin-LB1	SS	HPLC	GVVDILKGAAKDIAGHLASKVM-NH₂	2192.2	2191.2 (2191.1)	[60]
	AMP	Ocellatin-LB2	SS	HPLC	GVVDILKGAAKDIAGHLASKVMN-NH₂	2306.3	2305.0 (2304.9)	[60]
	NP	Caerulein	SOE	ACC	EQDY (HSO3) TGWMDF-NH2	-	-	[54,61]
	NP	Caerulein-like peptide	SOE	ACC	-	-	-	[40]
*Leptodactylus laticeps*	AMP	Ocellatin-L1	NI	HPLC	GVVDILKGAAKDLAGHLATKVMNKL-NH₂	25614.7	2206.3 (2206.3)	[62]
	AMP	Ocellatin-L2	NI	HPLC	GVVDILKGAAKDLAGHLATKVMDKL-NH₂	25624.6	2564	[63]
	AMP	Plasticin-L1	NI	HPLC	GLVNGLLSSVLGGGQGGGGLLGGIL	21642.2	2165.5	[63]
	NP	Caerulein	SOE	ACC	EQDY (HSO3) TGWMDF-NH₂	-	-	[54]
	NP	Caerulein-like peptide	SOE	ACC	-	-	-	[40]
*Leptodactylus latrans*	AMP	Ocellatin-1.1	EST	HPLC	GVVDILKGAGKDLLAH---------	16049.2	-	[64]
	AMP	Ocellatin-2.1	EST	HPLC	GVLDIFKDAAKQLIA----------	16009.2	-	[64]
	AMP	Ocellatin-3.1	EST	HPLC	GVLDILKNAAKNILA----------	15519.3	-	[64]
	AMP	Ocellatin-5	EST	HPLC	AVLDILKDVGKGLLSHFMEKV-NH₂	23113.0	2312.8	[64]
	AMP	Ocellatin-5.1	EST	HPLC	AVLDILKDVGKGLL-----------	14528.9	-	[64]
	AMP	Ocellatin-6	EST	HPLC	AVLDFIKAAGKGLVTNIMEKVG-NH₂	22732.8	2274.7	[64]
	AMP	Ocellatin-6.1	EST	HPLC	AVLDFIKAAGKGLVTNIM-------	18600.5	-	[64]
*Leptodactylus luctator*	AMP	Ocellatin-1	EST	HPLC	GVVDILKGAGKDLLAHLVGKISEKV-NH₂	25585.2	2559.1 (2560.0)	[65]
	AMP	Ocellatin-10	-	-	GLLDFLKAAGKGLVSNLIEKVG	2241.3	2184.8	[66]
	AMP	Ocellatin-11	-	-	GVLDIFKDAAKQILAHAAEKIG	2307.3	2250.8	[66]
	AMP	Ocellatin-2	EST	HPLC	GVLDIFKDAAKQILAHAAEKQI-NH₂	23783.3	2250.3 (2251.6)	[65]
	AMP	Ocellatin-3	EST	HPLC	GVLDILKNAAKNILAHAAEQI-NH₂	22012.5	2200.8 (2202.5)	[65]
	AMP	Ocellatin-4	EST	HPLC	GLLDFVTGVGKDIFAQLIKQI-NH₂	22743.0	2274.3 (2 274.2)	[67]
	AMP	Ocellatin-7	-	-	GVVDILKDTGKKLLSHLMEKIG	2393.4	2336.8	[66]
	AMP	Ocellatin-8	-	-	GVVDILKDTGKKLLSHLMEKVG	2379.4	2322.8	[66]
	AMP	Ocellatin-9	-	-	GVLDIFKDTGKKLLSHLMEKVG	2427.4	2370.8	[66]
	AMP	P1-Ll-1577	EST	LC	DEMKLDGFNMHLE-NH₂	15776.9	-	[68]
	AMP	P2-Ll-1298	EST	LC	AAGKGLVSNLLEK-NH₂	12987.6	-	[68]
	AMP	P3-Ll-2085	EST	LC	GLLDFLKAAGKGLVSNLLEK-NH₂	20852.2	-	[68]
*Leptodactylus macrosternum*	AMP	Ocellatin-C1	MS	HPLC	GILDFFKGPVKNALAE	1717.9	1718.2	[59]
	AMP	Ocellatin-C2	MS	HPLC	GLLGKGGLLAKVLA	13088.5	1310	[59]
*Leptodactylus nesiotus*	AMP	Ocellatin-1N	NI	HPLC	GAVVDILKGAGKNLLSLALNKLSEKV	2649.6	2649.3 (2649.6)	[58]
	AMP	Ocellatin-2N	NI	HPLC	GAVVDILKDTGKNLLSLALNKLSEKV	2737.6	2737.3 (2737.6)	[58]
	AMP	Ocellatin-3N	NI	HPLC	GIFDVLKNLAKGVITSLASa	1945.1	1945.1 (1945.3)	[58]
	AMP	Ocellatin-4N	NI	HPLC	GLFDVLKNLAKGVITSLASa	1945.1	1945.1 (1945.3)	[58]
*Leptodactylus pentadactylus*	AMP	Ocellatin-F1/Fallaxin	-	-	GVVDILKGAAKDIAGHLASKVMNKL-NH₂	25474.6	-	[69]
	AMP	Ocellatin-P1/ Pentadactylin	NI	HPLC	GLLDTLKGAAKNVVGSLASKVMELK-NH₂	25414.6	2540.5 (2540.5)	[70]
	NP	Caerulein	SOE	ACC	EQDY (HSO3) TGWMDF-NH₂	-	-	[54]
	NP	Caerulein-like peptide	SOE	ACC	-	-	-	[40]
	AMP	Ocellatin-PT1	EST	HPLC	GVFDIIKDAGKQLVAHAMGKIAEKV-NH₂	26374.7	2639.1	[18]
	AMP	Ocellatin-PT2	EST	HPLC	GVFDIIKDAGKQLVAHATGKIAEKV-NH₂	26074.7	2609	[18]
	AMP	Ocellatin-PT3	EST	HPLC	GVIDIIKGAGKDLIAHAIGKLAEKV-NH2	25285.1	2530	[18]
	AMP	Ocellatin-PT4	EST	HPLC	GVFDIIKGAGKQLIAHAMGKIAEKV-NH₂	2593.5	2595.1	[18]
	AMP	Ocellatin-PT5	EST	HPLC	GVFDIIKDAGRQLVAHAMGKIAEKV-NH₂	2665.5	2667.1	[18]
	AMP	Ocellatin-PT6	EST	HPLC	GVFDIIKGAGKQLIAHAMEKIAEKVGLNKDGN	3363.8	3365.9	[18]
	AMP	Ocellatin-PT7	EST	HPLC	GVFDIIKGAGKQLIAHAMGKIAEKVGLNKDGN	3291.8	3293.8	[18]
	AMP	Ocellatin-PT8	EST	HPLC	GVFDIIKGAGKQLIARAMGKIAEKVGLNKDGN	3310.9	3312.9	[18]
*Leptodactylus rhodonotus*	NP	Caerulein-like peptide	SOE	ACC	-	-	-	[40]
	NP	Caerulein	SOE	ACC	EQDY (HSO3) TGWMDF-NH₂	-	-	[54]
*Leptodactylus rugosus*	NP	Caerulein	SOE	ACC	EQDY (HSO3) TGWMDF-NH₂	-	-	[54]
*Leptodactylus stenodema*	NP	Caerulein	SOE	ACC	EQDY (SO3) TGWMDF-NH_2_	-	-	[54]
	NP	Caerulein-like peptide	SOE	ACC	-	-	-	[43]
	NP	Caerulein-like peptide	SOE	ACC	-	-		[40]
*Leptodactylus syphax*	AMP	Ocellatin-S1/ Syphaxin	EST	HPLC	GVLDILKGAAKDLAGHVATKVINKI	2543.5	-	[71]
*Leptodactylus validus*	AMP	Ocellatin-V1	NI	HPLC	GVVDILKGAGKDLLAHALSKLSEKV-NH₂	2560.5	2559.5 (2559.5)	[72]
	AMP	Ocellatin-V2	NI	HPLC	GVLDILKGAGKDLLAHALSKISEKV-NH₂	2574.5	2573.6 (2573.5)	[72]
	AMP	Ocellatin-V3	NI	HPLC	GVLDILTGAGKDLLAHALSKLSEKV-NH₂	2547.5	2546.5 (2546.5)	[72]
*Leptodactylus vastus*	AMP	Leptoglycin	EST	HPLC	GLLGGLLGPLLGGGGGGGGGLL	1761.0	1762	[73]
	AMP	Ocellatin-K1 (1-21)	EST	HPLC	GVVDILKGAAKDLAGHLASKV	2061.2	2062,44	[74]
	AMP	Ocellatin-K1(1-16)	EST	HPLC	GVVDILKGAAKDLAGH	1562.9	1563,82	[74]
*Physalaemus biligonigerus*	NP	Physalaemin	SOE	ACC	EADPDKFYGLM-NH₂	1284.6	-	[54,75,76]
	NP	Tachykinins	-	-	-	-	-	[61]
*Physalaemus centralis*	AMP	PEP1 _N4	EST	HPLC	GLKEFMKGLAKTALEHIAGALA	2268.3	2268.2 (2268.0)	[77]
	AMP	PEP2_N5	EST	HPLC	GLKEFMKGLAKTALEKIAGALA	2259.3	2259.3 (2259.1)	[77]
	AMP	PEP4_N6	EST	HPLC	GLKEFIKGLAKTALEKIAGALA	2241.3	2241.3 (2241.3)	[77]
	AMP	PEP5_N7	EST	HPLC	GLKEFMKDLAKTVVEKIAGALA	2331.3	2331.3 (2331.2)	[77]
	NP	Physalaemin	SOE	ACC	EADPDKFYGLM-NH_2_	1284.6	-	[54]
	NP	Tachykinins	-	-	-	-	-	[61]
*Physalaemus cuvieri*	NP	Physalaemin	SOE	ACC	EADPDKFYGLM-NH_2_	1284.6	-	[54]
*Physalaemus nattereri*	AMP	Nattererin-1	EST	HPLC	QPQPSFKNIVAGAIKVAAEKALNKIMDKLG-NH₂	3178.8	-	[78]
	AMP	Nattererin-2	EST	HPLC	QPQPSFRNIVAGAIKVAAEKALNKIMDKLG-NH₂	3206.8	-	[78]
	AMP	Ocellatin-1	EST	HPLC	GVVDILKGAGKDLLAHLVGKISEKV-NH₂	2558.5	-	[78]
	AMP	Ocellatin-3	EST	HPLC	GVLDILKNAAKNILAHAAEQI-NH₂	2201.3	-	[78]
	AMP	Ocellatin-5	EST	HPLC	AVLDILKDVGKGLLSHFMEKV-NH₂	2311.3	-	[78]
	NP	Physalaemin	SOE	ACC	EADPDKFYGLM-NH_2_	1284.6	-	[54]
	AMP	Antioxidin-I	EST	HPLC	TWYFITPYIPDK	1542.8	1543.69	[2]
	AMP	Nattererin-1	EST	HPLC	QPQPSFKNIVAGAIKVAAEKALNKIMDKLG-NH₂	3178.8	-	[79]
	AMP	Nattererin-2	EST	HPLC	QPQPSFRNIVAGAIKVAAEKALNKIMDKLG-NH₂	3206.8	-	[79]
	NP	(des-Arg9)-Bradykinin	EST	HPLC	RPPGFSPF		904.4 ( 904.5)	[79]
	NP	(Hyp3)-Bradykinin	EST	HPLC	RPHypGFSPFR		1076.5 (1076.6)	[79]
	NP	(Hyp3)-Bradykinin-VD	EST	HPLC	RPHypGFSPFRVD		1290.6 (1290.7)	[79]
	NP	(Hyp3, Thr6)-Bradykinin	EST	HPLC	RPHypGFTPFR		1090.5 (1090.6)	[79]
	NP	(Hyp3, Thr6)-Bradykinin	EST	HPLC	RPHypGFTPFRIY		1366.73 (1366.8)	[79]
	NP	(Thr6)-Bradykinin	EST	HPLC	RPPGFTPFR		1074.5 (1074.6)	[79]
	NP	(Thr6)-Phyllokinins	EST	HPLC	RPPGFTPFRIY		1350.73 (1350.8)	[79]
	NP	(Thr6, des-Arg9)-Bradykinin	EST	HPLC	RPPGFTPF		918.4 (918.54)	[79]
	NP	(Val1, Thr6)-Bradykinin	EST	HPLC	VPPGFTPFR		1017.5 (1017.6)	[79]
	NP	(Val1, Thr6)-Bradykinin-SPA	EST	HPLC	VPPGFTPFRSPA		1272.6 (1272.7)	[79]
	NP	(Val1, Thr6)-Bradykinin-VD	EST	HPLC	VPPGFTPFRVD		1231.6 (1231.7)	[79]
	NP	(Val1, Thr6, des-Arg9)-Bradykinin	EST	HPLC	VPPGFTPF		861.4 (861.5 )	[79]
	NP	Bradykinin	EST	HPLC	RPPGFSPFR		1060.5 (1060.6)	[79]
	NP	SO (Hyp3, Thr6)-Phyllokinins	EST	HPLC	RPHypGFTPFRIY(SO3H)		1446.6 (1446.7)	[79]
	NP	SO (Thr6)-Phyllokinins	EST	HPLC	RPPGFTPFRIY(SO3H)		1430.6 (1430.8)	[79]
*Physalaemus signifer*	NP	Physalaemin	SOE	ACC	EADPDKFYGLM-NH₂	1284.6	-	[54]
*Pleurodema somuncurense*	AMP	somuncurin-1	EST	HPLC	FIIWPLRYRK-NH₂	1390.8	1390.8	[80]
	AMP	somuncurin-2	EST	HPLC	FILKRSYPQYY-NH₂	1476.8	1476.8	[80]
	AMP	somuncurin-3	EST	HPLC	DDGEEEAESEEANPEENTEGEKKKKCRRRKGSKLLRRCRGVKI-NH₂	4986.5	4986.5	[80]
	AMP	somuncurin-4.1	EST	HPLC	TIYPLRSAE-NH₂	1048.6	1048.6	[80]
	AMP	somuncurin-4.2	EST	HPLC	YYQVSEERRRDLASLARLYALAR-NH₂	2798.5	2798.5	[80]
	AMP	somuncurin-4.2a	EST	HPLC	DLASLARLYALAR-NH₂	1431.8	1431.8	[80]
	AMP	somuncurin-4.3	EST	HPLC	NNEENELRRRVSFNRAVIHSLLG-NH₂	2722.4	2722.5	[80]
	AMP	somuncurin-4.3a	EST	HPLC	VSFNRAVIHSLLG-NH₂	1411.8	1411.8	[80]
	AMP	somuncurin-4.4	EST	HPLC	GIVSYHPRSSD-NH₂	1216.6	1216.6	[80]
	AMP	thaulin-3	EST	HPLC	NLVGSLLGGILKK-NH₂	1310.8	1310.8	[80]
	AMP	thaulin-Sl	EST	HPLC	DLLNGLLNPVLGIANGLTGGLVKK-NH₂	2388.4	2388.4	[80]
*Pleurodema thaul*	AMP	Gly-Thaulin-1	SY	HPLC	GNGNLLGGLLRPVLGVVKGLTGGLGKK	2586.6	-	[81]
	AMP	Thaulin-1	SY	HPLC	NGNLLGGLLRPVLGVVKGLTGGLGKK	2529.5	2531.08	[81]
	AMP	Thaulin-2	SY	HPLC	ELLGGLLDPVLGVANALTGGIIKK	2360.4	2361.85	[81]
	AMP	Thaulin-3	SY	HPLC	NLVGSLLGGILKK	1310.8	1311.63	[81]
	AMP	Thaulin-4	SY	HPLC	DDGEEAESEAANPEENTVGG	2018.8	2019.92	[81]

*: chromatographic technique applied for separation or purification of constituents. MW: Molecular weight. NP: Neuropeptide. AMP: Antimicrobial Peptides. ES: Electrical Stimulation. NI: Norepinephrine Injection. SOE: Solvent Extraction. MS: Manual Stimulation. SS: Skin Scraping. ACC: Alumina chromatography column. HPLC: High performance liquid chromatography. Mass expressed in Daltons: Real mass (Theoretical mass). a: Denotes C-terminal -amidation.

The peptide constituents of the skin secretions of Leptodactylidae species have an α-helical form, usually reported with an NH_2_ terminal. The most abundant peptides reported are ocellatins (Table 2). In addition, the most common C-terminal amino acids are glycine (G) or glycine-valine (GV) sequence, while N-terminal amino acids are glutamic acid (E), lysine (K), and valine (V). Glycines (G) seem to be a recurrent amino acid in multiple positions, some peptides are mainly constituted by G (e.g. leptoglycine, plasticin-L1, and Gly-Thaulin-1). Leucines (L) and lysines (Lys) are frequently observed in multiple positions.

There are two major categories of peptides in amphibian skin secretion: the neuroactive peptides (NP) and the antimicrobial peptides (AMPs) [82, 83]. The neuroactive peptides from Leptodactylidae are physalaemin (tachykinin family), bradykinins and their derivatives, caeruleins, and the caeruleins-like peptides (Mean = 1167.3 SD = 47.7; Max = 1446.6; Min = 861.4; n = 16) (Table 2).

Physalaemin, an NP, was reported from several *Physalaemus* species (Table 2). It exhibits positive effects in stimulating the intestine, ileum, duodenum, bladder, pancreas, and stomach, displaying intense hypotensive activity in mammals. Additionally, it can also induce saliva production and lacrimal secretion in several mammals and some birds [40, 41, 75, 83].

Bradykinin peptides are found in many anuran species, but they were recorded only in *Physalaemus* from the Leptodactylidae family. Bradykinins contain C-terminal COOH residues, and they are considered the main peptides reported from skin secretions of anurans [83, 84]. Bradykinins exhibit effects on smooth muscles, showing gastrointestinal effects in mammals, and they are also involved in the pain response, and potent immunostimulatory effects [44, 82, 84]. Only *Physalaemus nattereri* shows bradykinins in the family (Table 2, Figure). Barbosa et al. [79] described bradykinins by sequencing granular and inguinal glands from *P. nattereri* and observed the genes related to bradykinins are expressed more in inguinal glands, which may be related to behavioral defenses.

The peptide caerulein is a neuropeptide among the most studied in anurans (Table 2). This peptide and caerulein-like polypeptides are described in several *Leptodactylus* species. They have shown a stimulant effect on gastric and pancreas secretions, resulting in acute pancreatitis, being able to stimulate the musculature of the gut, except in the duodenum. Other effects of caerulein include the reduction of blood pressure at very low doses and sedative effects [44, 82]. Caerulein has also been reported as having potent analgesic properties with an effect 2,000 times higher than morphine [85]. This peptide has been described from *L. labyrinthicus, L. laticeps, L. pentadactylus, L. rhodonotus, L. rugosus,* and *L. stenodema* (Table 2, Figure 1).

Although Leptodactylidae has several neuroactive peptides in their skin secretion, a great diversity of antimicrobial peptides (AMPs) has been also described, highlighting the interest in this family for research of antimicrobial molecules. The AMPs have variations in molecular weight (Mean = 7449.8 SD = 957.3; Max = 26374.6; Min = 1048.5; n = 84) and number of amino acids (Table 2).

AMPs are grouped according to their structure as ⍺-helice, ꞵ-sheet, cyclic, and extended peptides, and they constitute the innate immunity system of several organisms, including plants, microorganisms, invertebrates, and vertebrates [86, 87]. Generally, these peptides are amphipathic molecules, containing hydrophobic residues and cationic properties [86, 87]. Due to their properties, AMPs can interact with bacteria membranes and induce a disturbance on its surface, leading to a loss of integrity or developing channels to increase the membrane permeability [86, 88, 89]. Additionally, some AMPs seem to be able to penetrate the bacteria membranes and influence metabolic processes, such as the synthesis of DNA, RNA, and proteins [87]. 

Several frog peptides, such as plasticin-1 and ocellatin-F1, have been described as solvent-dependent conformations by circular dichroism (CD) and Nuclear Magnetic Resonance (NMR) studies [72, 90, 91]. Plasticin-1, for example, shows a random coil conformation in water, β-sheet in methanol, and α-helical in the solvent trifluoroethanol and water 1:1 (v/v) [92]. The antimicrobial activity of peptides has been related to the complex interactions of factors that include their conformation (α-helicity), hydrophobicity, charge, and amphipathicity [93-95]. Ocellatin-F1 exhibits a strong correlation between its antimicrobial activity and the increase of hydrophobicity, the reduction of polar angles (measure of the amphipathic degree in an α-helical using the vector sum of hydrophobicities) is also correlated positively to the antimicrobial activities [72, 96]. AMPs of *Leptodactylus* species have the propensity to adopt an α-helical conformation in a membrane mimetic system [73], which is typical behavior for them, acquiring an active conformation in the membrane surface contact [60]. 

The first AMPs isolated in Leptodactylidae were the peptides ocellatins 1, 2, and 3, found in the secretion of *Leptodactylus ocellatus* (Table 2) [65]. In addition to ocellatins, other groups of AMPs described in Leptodactylidae were evaluated for a range of bacteria and fungi, as listed in Table 3. Generally, the studies with antimicrobial activity of the AMPs from anurans performed their sequencing and production by solid-phase peptide synthesis to expand the biological and pharmacological properties. Considering the potential antimicrobial of peptides, the minimum inhibitory concentration (MIC) lower than 30 µM are noticed for at least 18 peptides of Leptodactylidae, such as leptoglycin, nattererin-1, nattererin-2, ocellatin-5, ocellatin-6, ocellatin-F1, ocellatin-P1, ocellatin-S/Syphaxin (1-22), ocellatin-S (1-16), thaulin-1 and its derivative Gly-thaulin-1, P1-Ll-1577, P2-Ll-1298, P3-Ll-2085, PEP1_N4, PEP2_N5, PEP4_N6 and PEP5_N7. Among them, PEP4_N6 showed potent antimicrobial activity against the gram-negative *Escherichia coli* ATCC25922 (MIC = 2 µM) and *Klebsiella pneumoniae* ATCC 13883 (MIC = 2 µM), followed by PEP2_N5 and ocellatin-S/Syphaxin (1-22) with MIC of 4 µM for *E. coli* ATCC25922, besides PEP1_N4, PEP2_N5, and PEP5_N7 exhibited MIC of 4 µM for *K. pneumoniae*. These antimicrobial activities evidence the potential of anuran peptides, which demonstrated potent activities for gram-positive and gram-negative bacteria. For instance, ocellatin S (1-22), P3-Ll-208, and ocellatin-6 showed activity for gram-positive *Staphylococcus aureus* ATCC29213 with MIC values of 14.6, 15 and 28 µM, respectively. These results demonstrate that the studies of new antimicrobial peptides from skin sections of anurans are promising. 

**Table 3.** MIC values for microorganisms tested with peptides and extracts from the skin secretion of the Leptodactylidae family.

Species	Substance or extract	Pathogen	Gram	MIC (µM)	Reference
*Engystomops pustulosus*	Tigerinin-1EP	*Escherichia coli* ATCC 35218	Negative	>125 µM	[55]
		*Staphylococcus aureus* ATCC 12600	Positive	>125 µM	[55]
	pustulosin-1	*Escherichia coli* ATCC 35218	Negative	125 µM	[55]
		*Staphylococcus aureus* ATCC 12600	Positive	>125 µM	[55]
	pustulosin-3	*Escherichia coli* ATCC 35218	Negative	125 µM	[55]
		*Staphylococcus aureus* ATCC 12600	Positive	>125 µM	[55]
*Leptodactylus fallax*	LASP	*Escherichia coli*	Negative	-	[56]
		*Staphylococcus aureus*	Positive	-	[56]
	Ocellatin-F1/Fallaxin	*Batrachochytrium dendrobatidis*	-	100	[69]
		*Candida albicans* ATCC 90028	Positive	>160	[57]
		*Enterobacter cloacae* NHTCC 53001	Negative	20	[57]
		*Escherichia coli* ATCC 25922	Negative	40	[57]
		*Klebsiella pneumoniae* KK3 9904	Negative	80	[57]
		*Proteus mirabilis* ATCC 25933	Negative	>160	[57]
		*Pseudomonas aeruginosa* ATCC 27853	Negative	80	[57]
		*Staphylococcus aureus* NCTC 8325	Positive	>160	[57]
*Leptodactylus insularum*	Ocellatin-1I	*Enterococcus faecalis* ATCC 51299	Positive	>250	[58]
		*Enterococcus faecium* ATCC 19434	Positive	-	[58]
		*Escherichia coli* ATCC 35218	Negative	62.5	[58]
		*Klebsiella pneumoniae* ATCC 49472	Negative	125	[58]
		*Klebsiella pneumoniae* ATCC BAA-2814	Negative	>125	[58]
		*Pseudomonas aeruginosa* ATCC 27853	Negative	-	[58]
		*Salmonella typhimurium* ATCC 14028	Negative	250	[58]
		*Staphylococcus aureus* ATCC 12600* *	Positive	250	[58]
		*Staphylococcus aureus* ATCC BAA-2312* *	Positive	250	[58]
	Ocellatin-2I	*Enterococcus faecalis* ATCC 51299	Positive	>250	[58]
		*Enterococcus faecium* ATCC 19434	Positive	250	[58]
		*Escherichia coli* ATCC 35218	Negative	62.5	[58]
		*Klebsiella pneumoniae* ATCC 49472	Negative	125	[58]
		*Klebsiella pneumoniae* ATCC BAA-2814	Negative	125	[58]
		*Pseudomonas aeruginosa* ATCC 27853	Negative	>125	[58]
		*Salmonella typhimurium* ATCC 14028	Negative	125	[58]
		*Staphylococcus aureus* ATCC 12600* *	Positive	>250	[58]
		*Staphylococcus aureus*ATCC BAA-2312	Positive	>250	[58]
*Leptodactylus labyrinthicus*	Ocellatin-F1/Fallaxin	*Aggregatibacter actinomycetemcomitans* ATCC 29522	Negative	24.84	[60]
		*Candida lusitaniae* ATCC 56936	-	50.25	[60]
		*Escherichia coli* ATCC 25922	Negative	397.45	[60]
		*Staphylococcus aureus*ATCC 25923	Positive	109.91	[60]
	Ocellatin-LB1	*Aggregatibacter actinomycetemcomitans* ATCC 29522	Negative	222.37	[60]
		*Candida albicans* ATCC 18804	-	233.55	[60]
		*Candida lusitaniae* ATCC 56936	-	233.55	[60]
		*Escherichia coli* ATCC 25922	Negative	114.04	[60]
	Ocellatin-LB2	*Aggregatibacter actinomycetemcomitans* ATCC 29522	Negative	210.04	[60]
*Leptodactylus laticeps*	Ocellatin-L1	*Candida albicans* ATCC 90028	Positive	>200	[62]
		*Enterobacter cloacae* HNTCC 53001	Negative	50	[62]
		*Enterococcus faecalis* ATCC 29212	Positive	>200	[62]
		*Escherichia coli* ATCC 25922	Negative	50	[62]
		*Klebsiella pneumoniae* KK3 9904	Negative	100	[62]
		*Proteus mirabilis* ATCC 25933	Negative	>200	[62]
		*Pseudomonas aeruginosa* ATCC 27853	Negative	100	[62]
		*Staphylococcus aureus* NCTC 8325	Positive	>200	[62]
		*Staphylococcus epidermidis* RP62A	Positive	>200	[62]
	Ocellatin-L2	*Escherichia coli* ATCC 25726	Negative	>500	[62]
		*Staphylococcus aureus*ATCC 25923	Positive	>500	[62]
	Plasticin-L1	*Escherichia coli* ATCC 25726	Negative	>500	[62]
		*Staphylococcus aureus* ATCC 25923* *	Positive	>500	[62]
*Leptodactylus latrans*	Ocellatin-5	*Escherichia coli* ATCC 25922	Negative	64 µg/ml	[64]
		*Staphylococcus aureus*ATCC 29213	Positive	128 µg/ml	[64]
	Ocellatin-6	*Escherichia coli* ATCC 25922	Negative	32 µg/ml	[64]
		*Staphylococcus aureus*ATCC 29213	Positive	64 µg/ml	[64]
*Leptodactylus luctator*	Fraction >1kDa	*Bacillus cereus* DBFIQB28	Positive	-	[97]
		*Escherichia coli* DBFIQ Ec9	Negative	-	[97]
		*Mycobacterium tuberculosis* H37Rv	-	187.5 µg/mL	[97]
		*Pseudomonas sp* DBFIQ P 55	Negative	-	[97]
		*Staphylococcus aureus*DBFIQ S 21	Positive	-	[97]
	Fraction >2kDa	*Bacillus cereus* DBFIQB28	Positive	-	[97]
		*Escherichia coli* DBFIQ Ec9	Negative	-	[97]
		*Mycobacterium tuberculosis* H37Rv	-	NI	[97]
		*Pseudomonas sp* DBFIQ P 55	Negative	-	[97]
		*Staphylococcus aureus*DBFIQ S 21	Positive	-	[97]
	Methanol extract	*Bacillus cereus* DBFIQB28	Positive	-	[97]
		*Escherichia coli* DBFIQ Ec9	Negative	-	[97]
		*Mycobacterium tuberculosis* H37Rv	-	187.5 µg/mL	[97]
		*Pseudomonas sp* DBFIQ P 55	Negative	-	[97]
		*Staphylococcus aureus* DBFIQ S 21* *	Positive	-	[97]
	Ocellatin-1	*Escherichia coli* ATCC 25922	Negative	-	[65]
	Ocellatin-2	*Escherichia coli* ATCC 25922	Negative	-	[65]
	Ocellatin-3	*Escherichia coli* ATCC 25922	Negative	-	[65]
	Ocellatin-4	*Escherichia coli* ATCC 25922	Negative	64	[67]
		*Staphylococcus aureus* ATCC 29213	Positive	64	[67]
	P1-Ll-1577	*Escherichia coli* ATCC 25922	Negative	20	[68]
		*Staphylococcus aureus*ATCC 25923	Positive	40.5	[68]
	P2-Ll-1298	*Escherichia coli* ATCC 25922	Negative	24.6	[68]
		*Staphylococcus aureus*ATCC 25923	Positive	49	[68]
	P3-Ll-2085	*Escherichia coli* ATCC 25922	Negative	15	[68]
		*Staphylococcus aureus*ATCC 25923	Positive	15	[68]
	TAS	*Bacillus cereus* DBFIQB28	Positive	-	[97]
		*Escherichia coli* DBFIQ Ec9	Negative	-	[97]
		*Mycobacterium tuberculosis* H37Rv	-	NI	[97]
		*Pseudomonas sp* DBFIQ P 55	Negative	-	[97]
		*Staphylococcus aureus*DBFIQ S 21	Positive	-	[97]
*Leptodactylus macrosternum*	Fatty Extract	*Candida albicans* ICB 12	-	>1040	[98]
		*Candida krusei* ATCC 6258	-	512	[98]
		*Escherichia coli* ATCC 10532	Negative	>1040	[98]
		*Klebsiella pneumoniae* ATCC 4362	Negative	>1040	[98]
		*Pseudomonas aeruginosa* ATCC 15442	Negative	256	[98]
		*Staphylococcus aureus*ATCC 25923	Positive	>1040	[98]
*Leptodactylus nesiotus*	Ocellatin-1N	*Enterococcus faecalis* ATCC 51299	Positive	>250	[58]
		*Enterococcus faecium* ATCC 19434	Positive	250	[58]
		*Escherichia coli* ATCC 35218	Negative	62.5	[58]
		*Klebsiella pneumoniae* ATCC 49472	Negative	125	[58]
		*Klebsiella pneumoniae* ATCC BAA-2814	Negative	125	[58]
		*Pseudomonas aeruginosa* ATCC 27853	Negative	>125	[58]
		*Salmonella typhimurium* ATCC 14028	Negative	250	[58]
		*Staphylococcus aureus* ATCC BAA-2312* *	Positive	250	[58]
		*Staphylococcus aureus*ATCC 12600	Positive	250	[58]
	Ocellatin-3N	*Enterococcus faecalis* ATCC 51299	Positive	250	[58]
		*Enterococcus faecium* ATCC 19434	Positive	62.5	[58]
		*Escherichia coli* ATCC 35218	Negative	31.25	[58]
		*Klebsiella pneumoniae* ATCC 49472	Negative	62.5	[58]
		*Klebsiella pneumoniae* ATCC BAA-2814	Negative	62.5	[58]
		*Pseudomonas aeruginosa* ATCC 27853	Negative	62.5	[58]
		*Salmonella typhimurium* ATCC 14028	Negative	62.5	[58]
		*Staphylococcus aureus* ATCC 12600* *	Positive	31.25	[58]
		*Staphylococcus aureus*ATCC BAA-2312	Positive	31.25	[58]
*Leptodactylus pentadactylus*	Ocellatin-P1/ Pentadactylin	*Candida albicans* ATCC 90028	Positive	>200	[70]
		*Enterobacter cloacae* HNTCC 53001	Negative	50	[70]
		*Enterococcus faecalis* ATCC 29212	Positive	200	[70]
		*Escherichia coli* ATCC 25922	Negative	25	[70]
		*Klebsiella pneumoniae* KK3 9904	Negative	100	[70]
		*Proteus mirabilis* ATCC 25933	Negative	>200	[70]
		*Pseudomonas aeruginosa* ATCC 27853	Negative	100	[70]
		*Staphylococcus aureus* NCTC 8325	Positive	200	[70]
		*Staphylococcus epidermidis* RP62A	Positive	100	[70]
		*Streptococcus Group B* HNTCC 80130	Positive	50	[70]
*Leptodactylus pustulatus*	Ocellatin-PT1	*Escherichia coli* ATCC 25922	Negative	300	[18]
		*Klebsiella pneumoniae* ATCC 700603	Negative	>300	[18]
		*Salmonella choleraesuis* ATCC 14028	Negative	>300	[18]
		*Staphylococcus aureus*ATCC 29313	Positive	>300	[18]
	Ocellatin-PT2	*Escherichia coli* ATCC 25922	Negative	>310	[18]
		*Klebsiella pneumoniae* ATCC 700603	Negative	>310	[18]
		*Salmonella choleraesuis* ATCC 14028	Negative	>310	[18]
		*Staphylococcus aureus* ATCC 29313* *	Positive	>310	[18]
	Ocellatin-PT3	*Escherichia coli* ATCC 25922	Negative	320	[18]
		*Klebsiella pneumoniae* ATCC 700603	Negative	>320	[18]
		*Salmonella choleraesuis* ATCC 14028	Negative	>320	[18]
		*Staphylococcus aureus* ATCC 29313* *	Positive	>320	[18]
	Ocellatin-PT4	*Escherichia coli* ATCC 25922	Negative	80	[18]
		*Klebsiella pneumoniae* ATCC 700603	Negative	310	[18]
		*Salmonella choleraesuis* ATCC 14028	Negative	310	[18]
		*Staphylococcus aureus*ATCC 29313	Positive	>310	[18]
	Ocellatin-PT5	*Escherichia coli* ATCC 25922	Negative	300	[18]
		*Klebsiella pneumoniae* ATCC 700603	Negative	>300	[18]
		*Salmonella choleraesuis* ATCC 14028	Negative	>300	[18]
		*Staphylococcus aureus* ATCC 29313* *	Positive	>300	[18]
	Ocellatin-PT6	*Escherichia coli* ATCC 25922	Negative	120	[18]
		*Klebsiella pneumoniae* ATCC 700603	Negative	>240	[18]
		*Salmonella choleraesuis* ATCC 14028	Negative	>240	[18]
		*Staphylococcus aureus*ATCC 29313	Positive	>240	[18]
	Ocellatin-PT7	*Escherichia coli* ATCC 25922	Negative	60	[18]
		*Klebsiella pneumoniae* ATCC 700603	Negative	>240	[18]
		*Salmonella choleraesuis* ATCC 14028	Negative	240	[18]
		*Staphylococcus aureus*ATCC 29313	Positive	240	[18]
	Ocellatin-PT8	*Escherichia coli* ATCC 25922	Negative	60	[18]
		*Klebsiella pneumoniae* ATCC 700603	Negative	240	[18]
		*Salmonella choleraesuis* ATCC 14028	Negative	240	[18]
		*Staphylococcus aureus*ATCC 29313	Positive	240	[18]
*Leptodactylus syphax*	Syphaxin (1-16)	*Escherichia coli* ATCC 25922	Negative	10.6	[71]
		*Staphylococcus aureus* ATCC 29213	Positive	40.5	[71]
	Syphaxin (1-22)	*Escherichia coli* ATCC 25922	Negative	40.5	[71]
		*Staphylococcus aureus* ATCC 29213	Positive	14.6	[71]
*Leptodactylus validus*	Ocellatin-V1	*Escherichia coli* ATCC 25923	Negative	>200	[72]
		*Staphylococcus aureus* ATCC 25726* *	Positive	>200	[72]
	Ocellatin-V2	*Escherichia coli* ATCC 25923	Negative	>200	[72]
		*Staphylococcus aureus* ATCC 25726* *	Positive	>200	[72]
	Ocellatin-V3	*Escherichia coli* ATCC 25923	Negative	>200	[72]
		*Staphylococcus aureus* ATCC 25726* *	Positive	>200	[72]
	Fat-Extract	*Candida albicans* ICB 12	-	>1040	[98]
		*Candida krusei* ATCC 6258	-	256	[98]
		*Escherichia coli* ATCC 10532	Negative	>1040	[98]
		*Klebsiella pneumoniae* ATCC 4362	Negative	>1040	[98]
		*Pseudomonas aeruginosa* ATCC 15442	Negative	512	[98]
		*Staphylococcus aureus*ATCC 25923	Positive	>1040	[98]
	Leptoglycin	*Candida albicans* CEMM 01-3-075	-	>200	[73]
		*Candida tropicalis* CEMM 01-2-078	-	>200	[73]
		*Citrobacter freundii* ATCC 8090	Negative	75	[73]
		*Enterococcus faecalis* ATCC 29912	Positive	>200	[73]
		*Escherichia coli* ATCC 28922	Negative	50	[73]
		*Micrococcus luteus* ATCC 29912	Positive	>200	[73]
		*Microporum canis* CEMM 01-2-133	-	>200	[73]
		*Pseudomonas aeruginosa* ATCC 9027	Negative	8	[73]
		*Staphylococcus aureus*ATCC 25.923	Positive	>200	[73]
		*Trichophyton rubrum* CEMM0 1-1-100	-	>200	[73]
	Ocellatin-K1 (1-21)	*Escherichia coli* ATCC 25922	Negative	125 μg/ml	[74]
		*Staphylococcus aureus* ATCC 25923* *	Positive	NI	[74]
	Ocellatin-K1(1-16)	*Escherichia coli* ATCC 25922	Negative	125 μg/ml	[74]
		*Staphylococcus aureus* ATCC 25923* *	Positive	31.25μg/ml	[74]
*Physalaemus nattereri*	Antioxidin-I	*Enterococcus faecalis* ATCC 29212	Positive	256 µg/ml	[2]
		*Escherichia coli* ATCC 25922	Negative	>1024 µg/ml	[2]
		*Pseudomonas aeruginosa* ATCC 27853 ATCC 27853	Negative	>1024 µg/ml	[2]
		*Staphylococcus aureus*ATCC 25923	Positive	>1024 µg/ml	[2]
		*Escherichia coli* ATCC 25922	Negative	10	[79]
	Nattererin-2	*Escherichia coli* ATCC 25922	Negative	10	[79]
	PEP1_N4	*Candida albicans* ATCC 14053	-	>128	[77]
		*Escherichia coli* ATCC 25922	Negative	8	[77]
		*Klebsiella pneumoniae* ATCC 13883	Negative	4	[77]
		*Staphylococcus aureus* ATCC 25923	Positive	32	[77]
		*Staphylococcus epidermidis* ATCC 12228	Positive	64	[77]
	PEP2_N5	*Escherichia coli* ATCC 25922	Negative	4	[77]
		*Klebsiella pneumoniae* ATCC 13883	Negative	4	[77]
		*Staphylococcus aureus* ATCC 25923	Positive	64	[77]
		*Staphylococcus epidermidis* ATCC 12228	Positive	64	[77]
	PEP4_N6	*Candida albicans* ATCC 14053	-	>128	[77]
		*Escherichia coli* ATCC 25922	Negative	2	[77]
		*Klebsiella pneumoniae* ATCC 13883	Negative	2	[77]
		*Staphylococcus aureus* ATCC 25923	Positive	ND	[77]
		*Staphylococcus epidermidis* ATCC 12228	Positive	ND	[77]
	PEP5_N7	*Candida albicans* ATCC 14053	-	>128	[77]
		*Escherichia coli* ATCC 25922	Negative	32	[77]
		*Klebsiella pneumoniae* ATCC 13883	Negative	4	[77]
		*Staphylococcus aureus* ATCC 25923	Positive	ND	[77]
		*Staphylococcus epidermidis* ATCC 12228	Positive	128	[77]
	PEP2_N5	*Candida albicans* ATCC 14053	-	>128	[77]
*Pleurodema somuncurense*	somuncurin-1	*Escherichia coli* ATCC 25922	Negative	250µg/ml	[80]
		*Staphylococcus aureus*ATCC 29213	Positive	500µg/ml	[80]
	somuncurin-2	*Escherichia coli* ATCC 25922	Negative	600µg/ml	[80]
		*Staphylococcus aureus*ATCC 29213	Positive	>700 µg/ml	[80]
	somuncurin-4.2	*Escherichia coli* ATCC 25922	Negative	>700 µg/ml	[80]
		*Staphylococcus aureus*ATCC 29213	Positive	>700 µg/ml	[80]
	somuncurin-4.2a	*Escherichia coli* ATCC 25922	Negative	>700 µg/ml	[80]
		*Staphylococcus aureus* ATCC 29213* *	Positive	>700 µg/ml	[80]
	somuncurin-4.3	*Escherichia coli* ATCC 25922	Negative	>700 µg/ml	[80]
		*Staphylococcus aureus*ATCC 29213	Positive	>700 µg/ml	[80]
	somuncurin-4.3a	*Escherichia coli* ATCC 25922	Negative	>700 µg/ml	[80]
		*Staphylococcus aureus*ATCC 29213	Positive	>700 µg/ml	[80]
	thaulin-3	*Escherichia coli* ATCC 25922	Negative	600µg/ml	[80]
		*Staphylococcus aureus*ATCC 29213	Positive	>700 µg/ml	[80]
	thaulin-Sl	*Escherichia coli* ATCC 25922	Negative	>700 µg/ml	[80]
		*Staphylococcus aureus*ATCC 29213	Positive	>700 µg/ml	[80]
*Pleurodema thaul*	Gly-Thaulin-1	*Escherichia coli* ATCC 25922	Negative	62.5 µg/ml	[81]
		*Klebsiella pneumoniae* ATCC 700603	Negative	125 µg/ml	[81]
		*Staphylococcus aureus*ATCC 29213	Positive	500 µg/ml	[81]
	Thaulin-1	*Escherichia coli* ATCC 25922	Negative	62.5 µg/ml	[81]
		*Klebsiella pneumoniae* ATCC 700603	Negative	125 µg/ml	[81]
		*Staphylococcus aureus*ATCC 29213	Positive	500 µg/ml	[81]
	Thaulin-2	*Escherichia coli* ATCC 25922	Negative	NI	[81]
		*Klebsiella pneumoniae* ATCC 700603	Negative	NI	[81]
		*Staphylococcus aureus*ATCC 29213	Positive	NI	[81]
	Thaulin-3	*Escherichia coli* ATCC 25922	Negative	NI	[81]
		*Klebsiella pneumoniae* ATCC 700603	Negative	NI	[81]
		*Staphylococcus aureus*ATCC 29213	Positive	NI	[81]
	Thaulin-4	*Escherichia coli* ATCC 25922	Negative	NI	[81]
		*Klebsiella pneumoniae* ATCC 700603	Negative	NI	[81]
		*Staphylococcus aureus*ATCC 29213	Positive	NI	[81]

MIC values are presented in µM or µg/ml. NI: Non Inhibition.

Leptoglycin (MW: 1761.0) exhibited a MIC of 8 µM for the gram-negative *Pseudomonas aeruginosa*, while ocellatin-F1 (fallaxin) was only active in gram-negative *Enterobacter cloacae* (MIC = 20 µM) and *Aggregatibacter actinomycetemcomitans* (MIC = 25 µM). Although ocellatin-F1 shows potential activity only for two bacteria strains, it is relevant to notice that this peptide reveals a broad spectrum of action at concentrations lower than 110 µM against diverse gram-negative and positive bacteria and was active against pathogenic fungi (Table 3). Despite the high diversity of ocellatins, only six of them presented antimicrobial activity at concentrations lower than 30 µM which are the following: ocellatin S/Syphaxin (1-22) (MW = 2189.40 Da), ocellatin S (1-16) (MW = 1577.8 Da), ocellatin-5 (MW = 23113.0 Da), ocellatin-6 (MW = 22732.8 Da), ocellatin-P1 (MW = 26374.7 Da), and ocellatin-F1 (MW = 2547.5 Da) (Table 3). Peptides from the skin of Leptodactylidae species have similar inhibition of *E. coli* than ampicillin, azithromycin, cefotaxime, and nalidixic acid; all exhibited a MIC around 4 µM [86]. AMPs from Leptodactylidae with potential activity against *E. coli* were a fraction contained both nattererin-1, nattererin-2, the peptides ocellatin-5, ocellatin-6, ocellatin-P1, ocellatin S (1-16), Gly-thaulin-1, thaulin-1, P1-Ll-1577, P2-Ll-1298, and P3-Ll-208, which showed MIC varying between 10 to 28 µM (Table 3). 

In addition to the antimicrobial potentials represented by MIC values of peptides, they are also investigated concerning their hemolytic properties. Since the main mechanism of action of these peptides is the interaction with bacterial membranes, some of them can also affect the cellular membrane of mammals [99]. As a result, if a peptide shows a potent antimicrobial activity, but hemolysis of human erythrocytes and/or cytotoxicity in murine fibroblasts occurs at the concentration of MIC value, this peptide is considered poorly selective, and it can be rejected as a potential candidate for therapeutic application [99]. In this way, we can emphasize that most peptides from skin secretions of Leptodactylidae have reported no hemolytic effect, highlighting their selectivity [57, 60, 68, 70, 71, 73, 79, 81]. However, P3-Ll-2085, a mix of two other peptides, caused 100% hemolysis at 40 µM, which can limit the use of this molecule [68]. There is no information about the hemolytic properties of ocellatin-5 and ocellatin-6 [64]. 

Although several peptides reported from frog secretions have no antimicrobial activities for the human pathogenic microorganism strains evaluated [71, 86], it is important to highlight that wild microorganisms, in general, are more susceptible to the action of antimicrobial substances [60]. Also, it is common to find more than one type of peptide in the skin secretion of frogs that could present activity by synergistic effects, and they can be efficient in protecting the amphibian [89]. 

Therefore, beyond the active antimicrobial peptides from skin frogs, some peptides demonstrate low or absent antimicrobial properties but have shown selectivity for microorganisms. Additionally, these peptides can act by synergism or represent a change of permeability membrane when, in combination with antibiotics, assisting the access of the antibiotics into pathogenic microorganisms [18]. These appointments highlight the potential of peptides from skin frogs even for the peptides with low or absent antimicrobial properties, but future investigations are still required to understand them, including in vivo experiments. Additionally, the inactive peptides of frogs can be involved in other essential functions, such as amphibian survival or modulating the immune system response [53, 99]. 

## Origin and evolution of peptides in anurans

In anurans, the origins of peptides go back 150 million years [100] from a series of genes involved in other skin functions in front of a scenario of conquering new land environments and fulfilling all new necessities [82]. Evidence from Phyllomedusidae, Pelodryadidae, and Ranidae families show that encoding genes come from a large and unique family of genes with several duplication events resulting in an evolutionary divergence and producing more than 100.000 different peptides [100, 101]. Gene family is well conserved with origin from a common ancestor before the fragmentation of Gondwana during the late Jurassic and early Cretaceous, and they do not follow speciation [10, 100].

**Figure 3.**
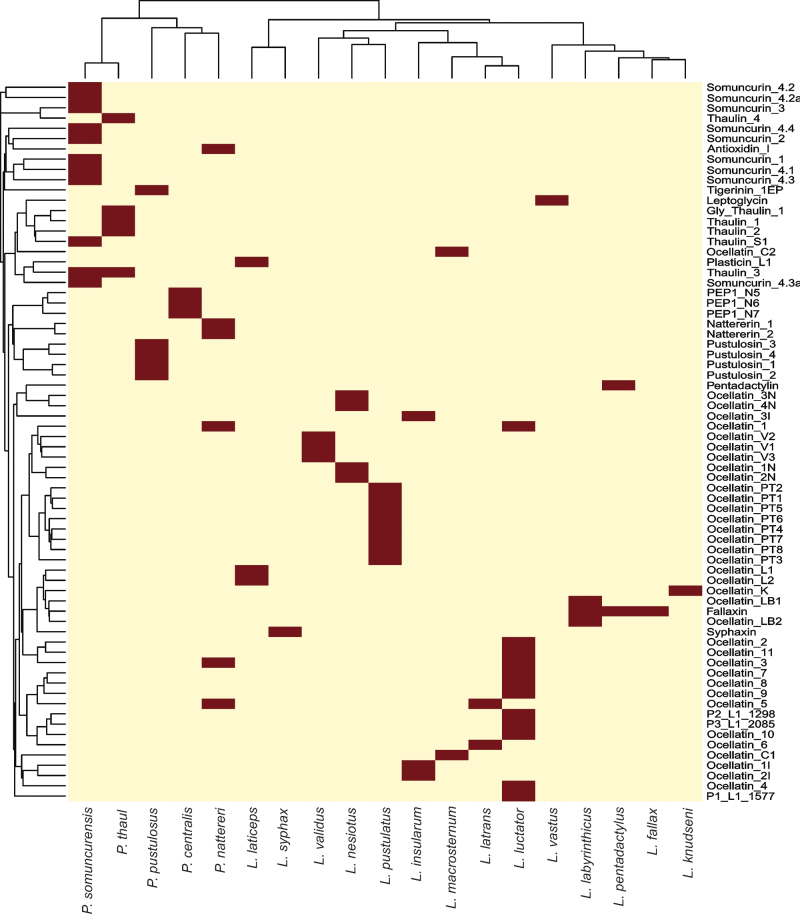
Heatmap representing the presence (brown) and absence of antimicrobial peptides in Leptodactylidae species. Phylogenetic tree of Leptodactylidae species (up) and ClustalW2 Phylogeny of the antimicrobial peptides (left).

Peptide-encoding genes display different mutation rates, even so, genes remain similar when compared to species phylogenetically distant [100]. Although conservative, peptides are rapid response systems for a faster pathogenic answer and depend on direct contact with pathogens, thus peptide encoding genes evolution does not follow speciation [82, 83]. We observed the same pattern when comparing a phylogenetic species tree with a ClustalW2 phylogeny of the antimicrobial peptides, where we can realize how little the peptides similarities reflected the phylogenetic relationship of the species (Figure 3). Caerulein, for example, is a peptide shared by several species from two species groups of *Leptodactylus* (*L. pentadactylus* and *L. fuscus*), which may indicate the origin of the peptide in a common ancestor of the group separation. Besides caerulein, ocellatin-F1 and ocellatin-K1 are the only peptides shared by the species of the *L. pentadactylus* group. The peptides of the *L. melanonotus* group are all exclusive, and no species share peptides. A similar situation occurs for Physalaemin, a peptide present in the skin of seven species from two different genera, and its origin must be a common ancestor of *Physalaemus* and *Engystomops*. Only two peptides are shared by *Physalaemus* and *Leptodactylus* (genus from different subfamilies), ocellatin-1 and ocellatin-3, both shared by *P. nattereri* and *L. luctator*. Sheared peptides have two possible explications; they can indicate an ancient origin previous to speciation or convergent evolution. 

Peptides exclusive for one species do not bring evolutionary information since they could either have an ancient origin that has been conserved until today by only one species or a recent origin that emerged after speciation. However, the first option seems less probable for species from the same groups. That is the case for most peptides, including ocellatins from *L. validus* and *L. pustulatus,* as well as *L. latrans, L. luctator,* and *L. macrosternum.* Another species with several exclusive peptides is *Pleurodema thaul*, but since there are no other studies with *Pleurodema* species, we cannot assure the exclusivity of these peptides. Despite all of the current knowledge, no phylogenetic comparative analyses are available, and genes involved in peptide productions remain unknown, as well as the mechanisms of expression.

## Ecological functions of skin secretions

Defensive secretion against predators can be classified as Odoriferous, Adhesive Noxious, and Slippery [102]. Additionally, these substances can have synergic actions with defensive behaviors, such as body-raising or thanatosis, to name a few [102]. For instance, *L. labyrinthicus* and *L. vastus* stretch the legs and lift the pelvis, while leaving the snout close to the ground, inguinal, and dorsal lateral skin presents bright colorations in red and yellow tones to a potential aggressor [78,103]. Besides the chemical defenses, the skin substances can act as cues and signals for many interactions including aggregation, territory defending, predator-prey interactions, mate attraction, and parental care [104, 105]. 

*Leptodactylus fallax* is a large frog from the Caribbean with restricted distribution [20]. Males are territorial and fight to defend the best call locations [106]. A peptide named *Leptodactylus* aggression-stimulating peptide (LASP) is used for males to stimulate other male aggressive behavior. This peptide has no action over females suggesting an exclusive agonist function [56].

*Lithodytes lineatus* is an Amazonian frog that can use the leaf-cutting ants' nest during reproduction without any consequences by mimicking ant chemical cues [107]. The leaf-cutting ants nest provides better environmental conditions to avoid egg drying and offers protection against terrestrial predators [107]. 

Multiple species of *L. latrans* and *L. melanonotus* groups display parental care behaviors, such as schooling guidance to sheltered places by pumping behavior (e.g. *L. insularum, L. podicipinus,* and *L. macrosternum*) [108-111]. Attending females call their tadpole schools by hitting the water with their pelvis to produce waves from a maximum distance of 18 cm. Consequently, schooling follows attending females through the ponds [110, 112]. Waves presumably transfer chemical signals that the tadpoles identify to follow attending females and to encourage tadpole schooling behavior [112, 113]. Inside the parental care context, the chemical signals and the biological mechanism remained unknown.

## Additional medicinal applications for the peptides of Leptodactylidae

In addition to the antibiotic activity, other applications are known for the secretions and peptides from the skin of amphibians, as well as for the secretions of Leptodactylidae species [53]. Biological and pharmacological applications of skin secretion from Leptodactylidae include immunomodulation, treatment of degenerative and zoonotic diseases, anticancer, antioxidant, and antifungal activities, control of arboviruses vectors, mosquito larvae control, and rabies control (Table 4) [2, 89, 91, 114, 115].

**Table 4.** Species of Leptodactylidae with pharmacological or biological properties.

Species name	Substance/Extract	Property	Reference
*Leptodactylus laticeps*	Plasticin-L1	Immunomodulatory	[91]
*Leptodactylus fallax*	Ocellatin-S1/ Syphaxin	Antiviral	[69]
*Leptodactylus knudseni*	crude secretion	Insecticidal	[114]
*Leptodactylus labyrinthicus*	Ocellatin-F1 and bufotenine	Anti-rabies	[115]
*Leptodactylus luctator*	Skin extract	Multi-target agents for Alzheimer Disease (AChE, MAOB) and DPPH	[116]
*Leptodactylus macrosternum*	Skin extract	Multi-target agents for Alzheimer Disease (BChE, MAOB) and DPPH	[116]
*Leptodactylus mystacinus*	Skin extract	Multi-target agents for Alzheimer Disease (MAOB)	[116]
*Leptodactylus pentadactylus*	Pentadactylin	Anti-proliferative	[117]
*Physalaemus nattereni*	Secretion	Anticancer	[118]
*Physalaemus nattereni*	Antioxidin-I	Antioxidant	[2]
*Physalaemus santafecinus*	Skin extract	Multi-target agents for Alzheimer Disease (AChE, BChE, MAOB) and DPPH	[116]
*Pseudopaludicula falcipes*	Skin extract	Multi-target agents for Alzheimer Disease (AChE, BChE, MAOB)	[116]

AChE: acetylcholinesterase; BChE: butyrylcholinesterase; MAOB: monoamine oxidase B.

One of the most relevant applications is cancer treatment. Pentadactylin from *Leptodactylus pentadactylus* and a crude secretion from *Physalaemus nattereri* (Figure 1) skin demonstrated a significant reduction of growth and proliferation of melanoma cells [118, 119]. Another application is on Alzheimer’s disease treatment, a neurodegenerative disorder of the brain and a major public health problem with 50 million cases worldwide [116, 120]. Extracts of *P. santafecinus,* and *P. falcipes* skin have shown inhibition of acetylcholinesterase, an enzyme that hydrolysis acetylcholine, which is a common factor associated with Alzheimer’s disease, and no haemolytic activity was observed for these extracts [116]. In addition, *Leptodactylus macrosternum* secretion shows antioxidant activity, which is associated with several diseases, including Alzheimer’s disease [116]

Plasticin-L1, a helical peptide rich in glycine and leucine from *L. laticeps*, has shown immunomodulatory properties since it stimulates cytokine production in macrophages from frog skin [91]. Immunomodulation was also reported for several amines listed in Table 1.

The compounds obtained from Leptodactylidae have also been evaluated to control virus vectors. Arboviruses, which are viruses transmitted through arthropods such as mosquitoes, are a major public health concern in tropical and subtropical countries, disseminating Dengue fever and resulting in over 100 million cases yearly [121, 122]. Therefore, the control of the Dengue vectors is crucial for the prevalence of tropical diseases [121]. *Aedes aegypti* is the main vector of Yellow Fever, Dengue, Chikungunya, and Zika [123], and *Anopheles darling* is the vector of malaria [124], two very important diseases in tropical countries. The crude skin secretion of *L. knudseni* exhibits insecticidal activity for *A. aegypti* and *A. darling*. The frog secretion affects adults and larvae of both species, and the ingestion of the secretion increases the dipterans mortality [114]. 

At least 16 species known of Rabies viruses are the cause of zoonotic neurotropic disease in mammals [125, 126]. Viruses attack and kill defensive T cells (lymphocytes) and stay in the nervous system, avoiding cell host apoptosis that results in encephalitic illness and posterior death [127]. Agency WHO estimates 59,000 rabies cases annually by dog-mediation, with higher prevalence in Asia and Africa [128]. In this manner, ocellatin-F1, a peptide found in *L. fallax*, *L. pentadactylus,* and *L. labyrinthicus* [57, 60, 70]*,* revealed antiviral activity against rabies virus [115]. Ocellatin-F1, in combination with bufotenine, an alkaloid from *Rhinella jimi*, showed synergistic activity in inhibiting viral penetration into BHK-21 cells, thereby restraining the infection [115]. These substances were also evaluated separately, and inhibitions lower than 25% were observed [115].

## Future considerations

Despite their high diversity and potential, only 9% of the species from the Leptodactylidae family were studied concerning chemical, biological, and pharmacological properties, which are relative to four genera (*Engystomops*, *Leptodactylus*, *Physalaemus,* and *Pleurodema*). This percentage is likely to decrease as the number of species in the family continues to grow, with nine species added to the family only in 2020, for example [20]. All the evaluated species belong to Leptodactyline and Leiuperine, and species of Paratelmatobiinae have not been studied yet. Therefore, there is a huge potential to be discovered from Leptodactylidae, as well as many ecological and evolutionary relationships to understand.

The OMICS techniques (e.g. proteomics, transcriptomics, and metabolomics) have provided opportunities for investigations more holistic from frog skin secretions [129]. These techniques combined with bioassays will allow better comprehension of the ecological issues and functionalities of the chemical signals and cues. Intra and interspecific frog communication are not limited to acoustic calls or visual signaling [129], instead chemical signaling plays several roles in social interaction like courtship, territoriality, and parental care, but this area has been underexplored in Leptodactylidae. 

RNA-seq analysis is another applicable technique with multiple advantages, allowing the identification of the entire transcriptomes and the quantification of the gene expression, making it possible for comparisons in particular scenarios such as stages of development, ecological situations, and/or environmental conditions [130]. Additionally, the rapid and harmless identification of alkaloids in poison frogs has been proved by the MasSpec Pen technique that applies mass spectrometry and represents an opportunity to discover new bioactive substances with an easy and fast method without sample preparation, since the data is obtained directly from tissue [131]

Leptodactylidae species reveal many antimicrobial peptides (AMPs) with potent activity against pathogenic bacteria. On the other hand, there is a significant number of species without any study, and highlights the potential source for new antimicrobial molecules from them. AMPs from Leptodactylidae species are majority cationic α-helical (positive charge +1 to +6 at pH 7) with hydrophobic amino acids (40 to 70%), being able to act by different mechanisms of action, presenting a broad spectrum of activities [87, 99]. Thus, these AMPs can interact with bacterial and fungal cell membranes and change, for example, the permeability, inducing the death of microorganisms [89, 99]. Since the AMPs act in cell membranes, which are highly conservated organs, it is difficult for pathogens to develop resistance against these substances [99]. Currently, antibiotic resistance is a worldwide public health issue [121]. This resistance is a natural process in which the microorganisms develop mechanisms to resist harmful substances from the environment as an adaptation to environmental pressure or threat [132]. Thus, the reach for new potent antibiotics to combat infections by clinical antibiotic resistance led traditional research to alternative sources such as animal species with natural exposure to pathogens like amphibians [1]. Natural exposure to pathogens, combined with diversity and live history, gives amphibians great potential to treat human diseases with skin secretion, an ecosystem service not well known [1, 16, 19].

## Conclusion

In summary, the current knowledge regarding the skin secretion of Leptodactylidae is limited compared to the family's diversity. The use of new technologies and reduced sample sizes for substance isolation and description is an advancement in the chemical studies of anuran skin. However, there are unstudied genera yet, as research focused on only the most common species.

The main compounds reported from Leptodactylidae are amines and peptides, mainly classified as neuropeptides and antimicrobial peptides. Ocellatins are the peptides most commonly reported. In addition, glycine (G) and glycine-valine (GV) are frequently observed as C-terminal amino acids, while N-terminal amino acids are observed as glutamic acid (E), lysine (K), and valine (V). The more active peptides against pathogenic bacterial strains (gram-positive and gram-negative) exhibit MIC of 1-15 µM, demonstrating the potential of Leptodactylidae species to search for new active compounds and stimulating the expansion of the investigation from them since they are scarcely explored. 

Although several peptides are potent antimicrobials, some inactive peptides could act in synergism, and they can also be combined with traditional antibiotics since they change the permeability of microbial membranes. These studies of the combinations (peptides and antibiotics) are relevant targets to investigate and develop new therapeutic strategies because they are unknown yet. Furthermore, these inactive antimicrobial peptides have been attributed to other ecological functions, including desiccation prevention, reproductive strategies, and the stimulation of aggressive behavior in male frogs. 

There are still gaps to fill in terms of ecological context, functions, and evolution. The origin of the encoded genes seems to be before Leptodactylidae divergence, as proved for other families, and there is no reason to believe that it could be different. However, these theories need to be proven for Leptodactylidae. Peptide gene evolution in the family remains unknown, and transcriptomic techniques represent an opportunity to understand this phenomenon.
